# Composite CA15-3, LDH, and Albumin Index as a Predictor of Survival in HER2-Positive Metastatic Breast Cancer Treated with Trastuzumab Emtansine

**DOI:** 10.3390/ph19060809

**Published:** 2026-05-22

**Authors:** Nagihan Kolkıran, Atike Pınar Erdoğan, Mustafa Şahbazlar, Müge Kurul Yeniay, Sinan Ünal, Mehmet Sinan Akarca, Elif Atağ Akyürek, Özge Demirkıran, Bilgin Demir, Ferhat Ekinci

**Affiliations:** 1 Department of Medical Oncology, Faculty of Medicine, Manisa Celal Bayar University, Manisa 45030, Turkey; nagihan.kolkiran@gmail.com (N.K.); m_sahbazlar@hotmail.com (M.Ş.); drferhatekinci@hotmail.com (F.E.); 2 Department of Medical Oncology, Faculty of Medicine, İzmir Katip Çelebi University Atatürk Training and Research Hospital, İzmir 35360, Turkey; mugekurul@gmail.com (M.K.Y.); drsinanunal@gmail.com (S.Ü.); 3 Department of Medical Oncology, Faculty of Medicine, Dokuz Eylül University, İzmir 35340, Turkey; akarcasinan@gmail.com (M.S.A.); elifatag@gmail.com (E.A.A.); 4 Department of Medical Oncology, Faculty of Medicine, Aydın Adnan Menderes University, Aydın 09100, Turkey; ozgedemrkrn@gmail.com (Ö.D.); bilgin287@hotmail.com (B.D.)

**Keywords:** breast cancer, trastuzumab emtansine, prognostic index, overall survival

## Abstract

**Background/Objectives**: Trastuzumab emtansine (T-DM1) is widely used in Human Epidermal Growth Factor Receptor2 (HER2)-positive metastatic breast cancer; however, outcomes vary, and reliable prognostic markers remain limited. We developed the CALA index as a composite biomarker integrating CA15-3, lactate dehydrogenase (LDH), and albumin. This study aimed to evaluate the prognostic value of the CALA index in metastatic breast cancer treated with T-DM1. **Methods**: This multicenter retrospective study included 168 patients treated with T-DM1 across four tertiary centers. The CALA index was calculated using pretreatment levels of CA15-3, LDH, and albumin. Receiver operating characteristic (ROC) curve analysis was used to determine the optimal cutoff value, and patients were stratified into groups accordingly. Survival outcomes and independent risk factors were assessed using Kaplan–Meier and Cox regression analyses. **Results**: The median overall survival (OS) was 26 months (95% CI: 21.3–30.7). ROC analysis identified an optimal CALA cutoff value of 118.3. Patients with CALA ≤ 118.3 demonstrated significantly longer OS compared with those with CALA > 118.3 (log-rank *p* = 0.006), with 1- and 3-year OS rates of 81.2% and 43.2% versus 69.8% and 22.7%, respectively. In univariate analysis, CALA > 118.3 was associated with worse OS (HR: 1.699; 95% CI: 1.151–2.506; *p* = 0.008), and this association remained significant in multivariate analysis (HR: 1.671; 95% CI: 1.088–2.565; *p* = 0.019). **Conclusions**: The CALA index was associated with overall survival in metastatic breast cancer treated with trastuzumab emtansine and may serve as a practical tool for risk stratification.

## 1. Introduction

Breast cancer remains the most commonly diagnosed malignancy worldwide and continues to represent a major cause of cancer-related mortality among women [1]. Although substantial advances in systemic therapies have improved survival outcomes, metastatic breast cancer is still characterized by considerable clinical heterogeneity, with markedly variable outcomes observed even among patients with similar clinicopathological features [2]. In HER2-positive metastatic breast cancer, the development of HER2-targeted therapies has significantly transformed the treatment landscape, leading to improved disease control and prolonged survival [3].

Trastuzumab emtansine, an antibody–drug conjugate combining the anti-HER2 activity of trastuzumab with a cytotoxic payload, has demonstrated superior efficacy compared with standard therapies in large randomized trials [3]. Notably, pivotal studies such as EMILIA and TH3RESA have established T-DM1 as a standard treatment option by significantly improving both progression-free and overall survival [3,4]. Despite these therapeutic advances, treatment outcomes remain heterogeneous, underscoring the need for robust, easily accessible biomarkers that can facilitate risk stratification and guide clinical decision-making [5]. Conventional prognostic factors, including tumor stage, receptor status, and performance status, provide valuable information but may not fully reflect the complex interactions between tumor biology and host-related factors [6].

Survival differences in cancer are influenced by both tumor burden and host-related factors, including systemic inflammation and nutritional status [7]. In this context, peripheral blood–based inflammatory markers and their derived indices, such as neutrophil–lymphocyte ratio (NLR), platelet–lymphocyte ratio (PLR), and other composite scores, have gained attention as potential prognostic tools in breast cancer [7]. Furthermore, integrated biomarkers incorporating inflammatory and nutritional components, including the HALP score and CRP-to-albumin ratio, have demonstrated independent associations with clinical outcomes [2,8]. More recently, studies focusing on patients receiving T-DM1 have demonstrated that inflammation-based indices, such as the pan-immune-inflammation value (PIV), are significantly associated with survival outcomes [9]. Lactate dehydrogenase is commonly used in clinical practice and generally reflects both tumor burden and metabolic activity, with higher levels often observed in patients with poorer outcomes [10]. Serum albumin, on the other hand, reflects both nutritional and inflammatory status, and decreased levels have been associated with poorer survival in cancer patients [8].

Tumor markers such as CA15-3 are frequently elevated in metastatic breast cancer and correlate with disease burden, although their independent prognostic value remains debated [11]. However, most existing studies have evaluated these biomarkers individually or within inflammation-focused models, while integrative approaches combining tumor burden, metabolic activity, and host-related factors remain limited in this specific patient population [9].

In clinical practice, currently available markers do not always reflect both tumor-related characteristics and the patient’s overall condition simultaneously [6]. Because CA15-3, LDH, and albumin represent complementary aspects of tumor biology and host status, combining these biomarkers may provide a more comprehensive prognostic assessment than evaluating each parameter individually. In this context, we developed a novel composite biomarker, termed the CALA index, calculated as (CA15-3 × LDH)/albumin. The aim of this study was to evaluate the prognostic significance of the CALA index in patients with HER2-positive metastatic breast cancer treated with T-DM1.

## 2. Results

### 2.1. Patient Demographics and Clinical Characteristics

**Figure 1:**
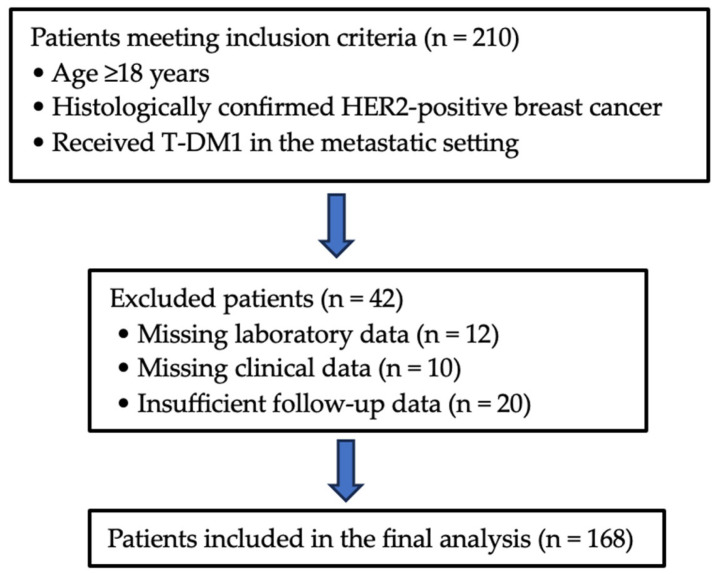
Flowchart of patient selection and study cohort formation.

A total of 210 HER2-positive metastatic breast cancer patients treated with T-DM1 were assessed for eligibility. After the exclusion of 42 patients because of missing laboratory data, insufficient follow-up data, or incomplete clinical records, 168 patients were included in the final analysis. The patient selection process is shown in the flowchart in Figure 1. The median age was 55.5 years (range, 26–85), with a median body mass index of 26.7 kg/m^2^. Most patients had an ECOG performance status of 0–1 (91.7%), and 51.8% had stage IV disease at diagnosis.

Estrogen and progesterone receptor positivity were observed in 82.8% and 64.6% of patients, respectively, while HER2 expression was scored as (+3) in 76.0%. Invasive ductal carcinoma was the predominant histological subtype (94.6%), and the median Ki-67 value was 30%. Metastatic involvement most commonly included bone (60.1%), followed by liver (41.9%) and lung (39.9%), with brain metastases observed in 22.6% of patients. De novo metastatic presentation was slightly more frequent than recurrence (53.0% vs. 47.0%).

T-DM1 was administered as second-line therapy in 50.0% of patients and as third-line therapy in 27.4%. Prior trastuzumab exposure was documented in 93.5% of patients and pertuzumab in 54.8%. Treatment response was distributed across partial response (40.7%), stable disease (21.6%), and progressive disease (26.9%), while a complete response was observed in 10.8% of patients.

**Figure 2:**
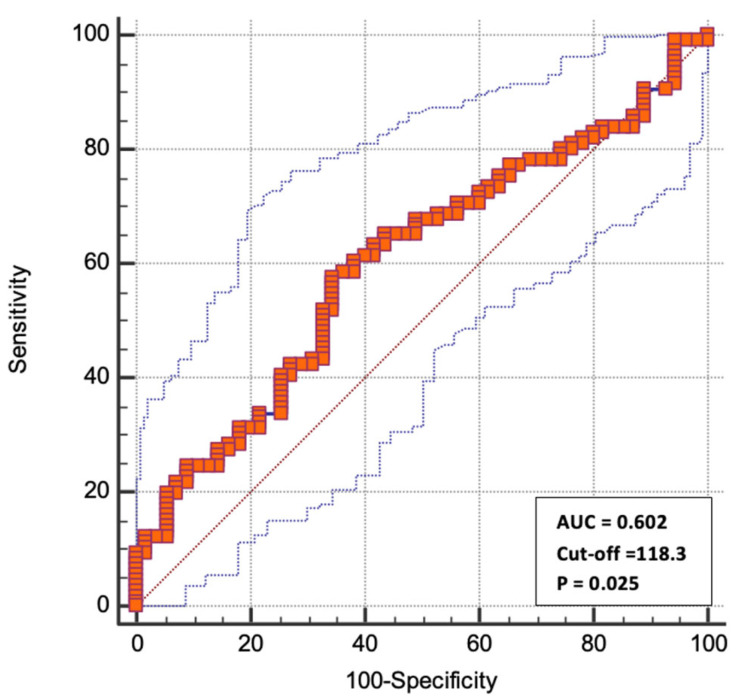
ROC curve analysis of the CALA index for overall survival prediction. Orange line: ROC curve; red diagonal line: reference line; blue dashed lines: 95% confidence interval. AUC: area under the curve.

ROC analysis identified a CALA cutoff value of 118.3, with an area under the curve (AUC) of 0.602 (*p* = 0.025), as shown in Figure 2. Patients were subsequently categorized into low (≤118.3) and high (>118.3) CALA groups. Between-group comparisons showed higher rates of liver metastases (58.5% vs. 28.2%, *p* < 0.001) and bone metastases (68.3% vs. 50.6%, *p* = 0.022) in patients with elevated CALA values. CRP (*p* = 0.038), LDH (*p* < 0.001), and CA15-3 levels (*p* < 0.001) were also higher in this group. In addition, T-DM1 was administered in later lines more frequently (*p* = 0.035), and the number of treatment lines was higher (*p* = 0.006). Detailed baseline characteristics and between-group comparisons are provided in Table 1. Regarding treatment safety, most adverse events were grade 1–2 in severity, while grade 3–4 toxicities were relatively uncommon. The most frequently observed adverse events included myalgia (20.4%), thrombocytopenia (13.8%), and neutropenia (10.8%). No statistically significant differences in adverse event profiles were identified between the low- and high-CALA groups (Table 2).

### 2.2. Univariate and Multivariate Analyses of Overall Survival and Progression-Free Survival

In univariate analysis for overall survival, the presence of liver metastasis (HR: 2.047, 95% CI: 1.397–2.999, *p* < 0.001), brain metastasis (HR: 1.752, 95% CI: 1.164–2.637, *p* = 0.007), and bone metastasis (HR: 1.527, 95% CI: 1.032–2.259, *p* = 0.034) each emerged as risk factors for shorter OS. Higher levels of CRP (HR, 1.009; *p* = 0.034) and LDH (HR, 1.002; *p* = 0.001) were also associated with increased mortality risk, whereas elevated albumin appeared protective (HR, 0.936; *p* = 0.003). When examined as a continuous measure, each one-unit increment in the CALA index was linked to poorer OS (HR, 1.271; 95% CI, 1.099–1.471; *p* = 0.001). Using a cutoff of 118.3, patients in the high-CALA group had significantly shorter OS compared to those in the low-CALA group (HR, 1.699; 95% CI, 1.151–2.506; *p* = 0.008). On multivariate analysis, CALA > 118.3 remained an independent risk factor for OS (HR, 1.671; 95% CI, 1.088–2.565; *p* = 0.019). Lung metastasis (HR, 1.509; 95% CI, 1.012–2.250; *p* = 0.044) and elevated CRP (HR, 1.008; 95% CI, 1.000–1.016; *p* = 0.045) were also identified as predictors of shorter OS. Patients who developed grade 1–2 adverse events had better overall survival (HR, 0.515; 95% CI, 0.331–0.803; *p* = 0.003). Detailed results are shown in Table 3.

For PFS, several factors were associated with disease progression. A higher number of treatment lines (HR, 1.164; 95% CI, 1.072–1.265; *p* < 0.001), elevated CRP (HR, 1.008; *p* = 0.040), higher LDH (HR, 1.001; *p* = 0.048), and each one-unit increase in CALA (HR, 1.236; 95% CI, 1.084–1.409; *p* = 0.002) predicted shorter PFS. Using the same cutoff of 118.3, patients in the high-CALA group had a higher risk of progression compared to those in the low-CALA group (HR, 1.401; 95% CI, 1.004–1.956; *p* = 0.047). In multivariate analysis, the number of prior treatment lines remained a predictor of disease progression (HR, 1.151; 95% CI, 1.047–1.267; *p* = 0.004), whereas CALA > 118.3 was no longer significantly associated with PFS after adjustment (HR, 1.112; 95% CI, 0.767–1.613; *p* = 0.576). These results are presented in Table 4.

### 2.3. Survival Outcomes

The median follow-up duration was 53 months. Overall survival in the study population reached a median of 26 months (95% CI, 21.3–30.7). One-year OS was 75.2%, dropping to 53.8% at two years and 34.4% at three years, while at five and seven years, OS rates were 21.4% and 17.7%, respectively.

**Figure 3:**
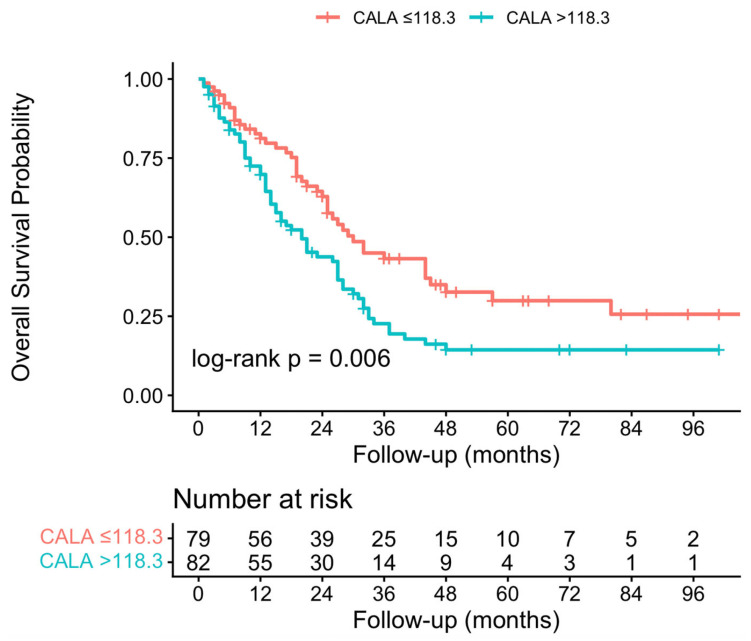
Kaplan–Meier curve for overall survival according to the CALA group.

Patients were then divided into two groups based on the CALA cutoff of 118.3, and the log-rank test revealed a significant difference in OS between them, as shown in Figure 3 (*p* = 0.006). The low-CALA group had higher survival rates at all time points: 81.2% vs. 69.8% at one year, 43.2% vs. 22.7% at three years, and 29.9% vs. 14.4% at five years.

**Figure 4:**
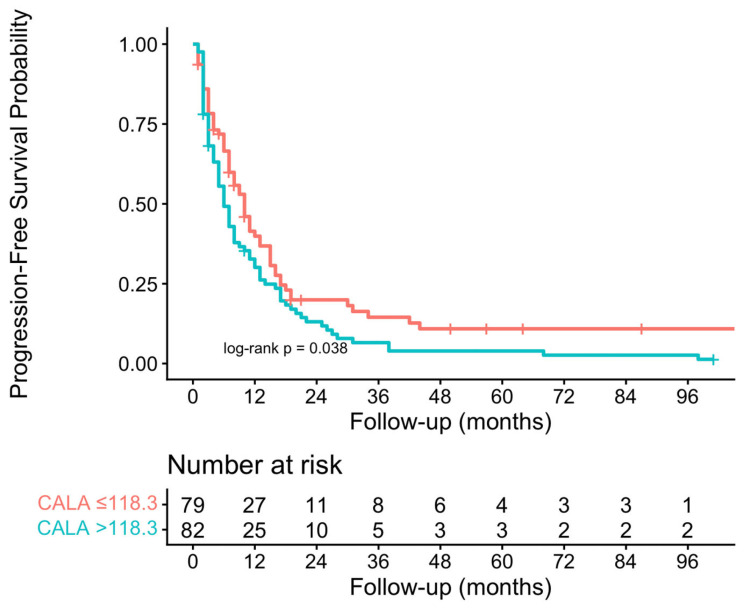
Kaplan–Meier curve for progression-free survival according to the CALA group.

Median PFS for the study population was 8 months (95% CI, 6.2–9.8). The 1-, 2-, and 3-year PFS rates were 35.4%, 16.9%, and 10.9%, respectively, while the five- and seven-year PFS rates were 7.3% and 5.8%. Using the same CALA cutoff, the log-rank test showed a significant difference in PFS between the two groups (*p* = 0.038) (Figure 4). The low-CALA group had better PFS at each time point: 42.2% vs. 30.9% at one year, 17.1% vs. 7.2% at three years, and 12.2% vs. 4.3% at five years.

### 2.4. Comparison of Prognostic Performance

CALA showed better discriminative ability than CA15-3, albumin, and LDH at each evaluated time point. The AUC values for CALA were 0.686, 0.758, 0.758, 0.772, and 0.764 at 12, 24, 36, 48, and 60 months, respectively (mean AUC 0.748). In comparison, CA15-3 showed lower overall discrimination (mean AUC 0.739), while albumin and LDH demonstrated more limited performance (mean AUC 0.734 and 0.728, respectively). In terms of model fit, CALA yielded the lowest Akaike Information Criterion (AIC) value (884.6), followed by CA15-3 (889.1), whereas albumin (937.6) and LDH (938.4) showed substantially higher AIC values. Although all models were statistically significant based on likelihood ratio testing, CALA provided the most consistent prognostic performance.

## 3. Discussion

Trastuzumab emtansine has been evaluated in both clinical trials and real-world settings in patients with HER2-positive metastatic breast cancer [3,12]. While clinical trials have demonstrated its efficacy, observational data indicate that treatment outcomes may differ across patient populations treated in routine practice [3,12]. This variation suggests that conventional clinicopathological characteristics alone may not be sufficient to fully capture differences in survival. Therefore, there is a continued need for additional biomarkers that better reflect disease biology and improve risk stratification.

In this study, we evaluated the prognostic value of a composite biomarker integrating CA15-3, lactate dehydrogenase, and albumin in patients with metastatic breast cancer treated with T-DM1. The findings indicate that this index is associated with overall survival, supporting its potential role in risk stratification.

The prognostic significance of tumor markers such as CA15-3 has been well established, particularly in metastatic breast cancer, where elevated levels reflect tumor burden and disease activity [13]. However, the independent prognostic value of CA15-3 remains controversial, as its association with survival may be influenced by other clinical and laboratory variables in multivariable analyses [11,14]. These findings suggest that tumor burden alone may not be sufficient to fully explain differences in survival and highlight the limitations of relying on a single biomarker.

Lactate dehydrogenase has emerged as a marker of tumor metabolic activity and aggressiveness. Elevated LDH levels are associated with increased glycolysis, tumor proliferation, and immune evasion, all of which contribute to adverse outcomes [15]. In metastatic breast cancer, LDH has been shown to be associated with survival and may improve prognostic accuracy when combined with other biomarkers [15]. Meta-analyses across solid tumors have consistently shown that high LDH is associated with poorer survival, reinforcing its role as a key prognostic indicator [16]. Elevated LDH levels may also reflect increased tumor burden and aggressive disease biology rather than intrinsic treatment resistance [16], which should be considered when interpreting the prognostic significance of the CALA index.

Albumin, another component of the index, represents both nutritional status and systemic inflammation. Hypoalbuminemia is associated with cancer-related inflammation, impaired immune response, and worse survival outcomes [17]. Hypoalbuminemia may also reflect chronic systemic inflammation, cancer-related cachexia, impaired hepatic protein synthesis, and reduced physiological reserve, all of which may contribute to poorer treatment tolerance and adverse clinical outcomes [17]. Accordingly, composite indices incorporating albumin, such as the HALP score, have demonstrated significant prognostic value in breast cancer [18]. Similarly, the CALLY index, which integrates CRP, albumin, and lymphocyte count, has been shown to reflect inflammatory, nutritional, and immune status simultaneously, with lower values associated with tumor progression and reduced survival [19].

Previous studies evaluating prognostic markers in HER2-positive metastatic breast cancer have primarily focused on hematologic inflammatory indices. Low baseline NLR has been associated with prolonged progression-free survival and overall survival in HER2-positive metastatic breast cancer patients treated with T-DM1, whereas elevated derived neutrophil to lymphocyte ratio (dNLR) has been linked to inferior survival outcomes [15,20]. These findings highlight the prognostic relevance of systemic inflammation in HER2-positive metastatic breast cancer. Although CALA is not a hematologic inflammatory index, higher CALA levels were likewise associated with poorer survival in our T-DM1-treated cohort. Unlike NLR or dNLR, which are derived solely from peripheral blood cell counts, CALA incorporates CA15-3, LDH, and albumin within a single composite biomarker.

Composite biomarker models suggest that combining multiple parameters may improve prognostic performance compared with single markers. A prognostic model integrating LDH, CRP, CA15-3, and CA125 demonstrated improved predictive accuracy in metastatic breast cancer [21]. Among composite models evaluated in HER2-positive metastatic breast cancer, a lower baseline systemic immune–inflammation index was associated with improved PFS and OS in patients receiving trastuzumab-based therapy [22]. In line with these findings, patients with high CALA levels also had poorer outcomes in our cohort. Unlike studies conducted in broader HER2-positive metastatic breast cancer populations, our study focused on a more homogeneous cohort of T-DM1-treated patients.

In T-DM1-treated HER2-positive metastatic breast cancer patients, PIV, a composite index derived from neutrophil, platelet, monocyte, and lymphocyte counts, was associated with significantly inferior PFS and OS [9]. Similarly, prognostic nutritional index (PNI) and albumin-to-alkaline phosphatase ratio (AAPR) were also shown to be associated with survival outcomes in patients receiving T-DM1, with low PNI significantly associated with poorer overall survival and low AAPR associated with inferior overall and progression-free survival [23]. In line with these findings, elevated CALA values in our cohort were associated with significantly worse survival outcomes. However, unlike PIV, PNI, and AAPR, CALA incorporates both tumor-related and host-related parameters.

The composite model developed in the present study combines tumor burden (CA15-3), metabolic activity (LDH), and systemic inflammatory and nutritional status (albumin) within a single biomarker. Stratification according to the CALA index demonstrated significant differences in overall survival between risk groups. In addition, its performance was compared with those of its individual components, including CA15-3, LDH, and albumin, using time-dependent AUC and AIC analyses. Although the observed differences were modest, our model showed more consistent discriminative performance across the evaluated time points. Furthermore, the multicenter real-world design may better reflect routine clinical practice. The use of routinely available laboratory parameters may also support potential clinical use.

Despite the strengths of the multicenter design and the real-world nature of the cohort, certain limitations should be considered when interpreting the findings of this study. Due to the retrospective design, an inherent risk of selection bias cannot be excluded. Also, the sample size remained relatively limited, restricting the ability to perform subgroup-specific ROC analyses and determine distinct cutoff values according to metastatic sites, as well as detailed subgroup analyses. Heterogeneity in the treatment line of T-DM1 administration among patients may also reflect evolving treatment practices, reimbursement policies, and physician preferences over time. Inflammatory or hepatic comorbidities that could potentially influence LDH or albumin levels were not specifically excluded because of the retrospective real-world design of the study. In addition, the optimal cutoff value for the CALA index was determined using ROC curve analysis within the study cohort and was not externally validated. Although the CALA index remained significantly associated with overall survival in multivariable analysis, the findings related to progression-free survival showed more borderline statistical significance and should be interpreted cautiously. Future large-scale prospective studies and independent external validation cohorts are needed to further evaluate the reliability and utility of the CALA index before routine clinical implementation.

## 4. Materials and Methods

### 4.1. Participants

This study was designed as a retrospective, multicenter analysis including patients with metastatic breast cancer who were treated with T-DM1. Data were collected from four tertiary oncology centers in Turkey: Manisa Celal Bayar University, İzmir Katip Çelebi University Atatürk Training and Research Hospital, Dokuz Eylül University, and Aydın Adnan Menderes University. Patients aged 18 years or older with histologically confirmed breast cancer who received T-DM1 in the metastatic setting between September 2016 and October 2025 were eligible for inclusion. Cases with missing clinical information, unavailable laboratory data, or insufficient follow-up data were excluded from the analysis. Clinical and treatment-related data were obtained from institutional records, including demographic characteristics, disease features, treatment details, and pretreatment laboratory parameters such as LDH, albumin, and CA15-3. All laboratory measurements were obtained within 14 days before the initiation of T-DM1 treatment.

### 4.2. Composite Index Calculation

The CALA index was calculated using the following formula: CALA = (CA15-3 × LDH)/albumin. ROC curve analysis was performed to determine the optimal cutoff value of the CALA index for overall survival prediction. The optimal threshold was identified according to the maximum Youden index, and the CALA cutoff value was determined as 118.3 (Youden index = 0.230, AUC = 0.602, *p* = 0.025). Patients were subsequently stratified into low- and high-CALA groups according to the identified cutoff value.

### 4.3. Survival Analysis

The primary survival endpoints of the study were overall survival and progression-free survival (PFS). Overall survival was calculated from the start date of T-DM1 therapy to death from any cause or last follow-up. Progression-free survival was defined as the interval between T-DM1 initiation and documented disease progression or death. Survival probabilities were estimated using the Kaplan–Meier method, and survival differences between groups were compared using the log-rank test. Kaplan–Meier survival curves and number-at-risk plots were generated using R software version 4.5.3 (R Foundation for Statistical Computing, Vienna, Austria).

### 4.4. Statistical Analyses

All statistical analyses were carried out using IBM SPSS Statistics version 27.0 (International Business Machines Corporation, Armonk, NY, USA). Categorical data were summarized using frequencies and percentages, whereas continuous variables were expressed using mean, standard deviation, median, and range values. Differences between groups were evaluated using the chi-square test for categorical variables and the Mann–Whitney U test for continuous variables due to non-normal distribution. Cox proportional hazards regression analyses were performed to evaluate factors associated with survival outcomes. Variables considered clinically or statistically relevant were included in univariable and multivariable analyses. Proportional hazard assumptions were assessed using log-survival plots before the Cox regression analyses. A *p*-value below 0.05 was considered statistically significant.

## 5. Conclusions

In conclusion, the CALA index was associated with overall survival in patients with HER2-positive metastatic breast cancer treated with T-DM1. In our cohort, higher CALA values were associated with poorer survival outcomes. Given its simple calculation based on routinely available laboratory parameters, the index may have potential prognostic relevance in this setting. However, further prospective studies and independent external validation cohorts are needed before its clinical applicability can be more clearly established.

## Figures and Tables

**Table 1:** Demographic and clinical characteristics of patients.

Variables		Total n (%)	CALA ≤ 118.3 n (%)	CALA > 118.3 n (%)	*p*-Value
Age	Median (Min–Max)	55.50 (26–85)	55 (26–85)	55.5 (30–81)	0.870
BMI	Median (Min–Max)	26.70 (17–169)	27.24 (20–169)	26.72 (19–50)	0.460
ECOG	0	112 (66.7)	56 (70.9)	51 (62.2)	0.549
	1	42 (25.0)	17 (21.5)	23 (28.0)	
	2	13 (7.7)	6 (7.6)	7 (8.5)	
	3	1 (0.6)	0 (0.0)	1 (1.2)	
Menopausal Status	Premenopausal	68 (41.2)	30 (38.5)	33 (41.2)	0.720
	Postmenopausal	97 (58.8)	48 (61.5)	47 (58.8)	
Stage at diagnosis	1	12 (7.1)	4 (5.1)	8 (9.8)	0.425
	2	37 (22.0)	20 (25.3)	16 (19.5)	
	3	32 (19.0)	17 (21.5)	13 (15.9)	
	4	86 (51.8)	38 (48.1)	45 (54.9)	
Metastatic Pattern	De novo metastasis	89 (53.0)	37 (46.8)	48 (58.5)	0.137
	Recurrence	79 (47.0)	42 (53.2)	34 (41.5)	
Tumor localization	Right	66 (39.3)	39 (49.4)	25 (30.5)	0.043
	Left	95 (56.5)	38 (48.1)	54 (65.9)	
	Bilateral	7 (4.2)	2 (2.5)	3 (3.7)	
ER status	Positive	106 (82.8)	106 (82.8)	71 (84.5)	0.216
	Negative	22 (17.2)	22 (17.2)	13 (15.5)	
PR status	Positive	73 (64.6)	73 (64.6)	42 (60.9)	0.528
	Negative	40 (35.4)	40 (35.4)	27 (39.1)	
Ki-67	Median (Min–Max)	30 (1–95)	30 (1–95)	30 (2–90)	0.388
HER2 status	HER2 (+2)	40 (24.0)	18 (23.1)	21 (25.6)	0.709
	HER2 (+3)	127 (76.0)	60 (76.9)	61 (74.4)	
Histologic subtype	Invasive ductal	159 (94.6)	75 (94.9)	78 (95.1)	0.407
	Invasive lobular	4 (2.4)	1 (1.3)	3 (3.7)	
	Other	5 (3.0)	3 (3.8)	1 (1.2)	
Liver metastasis		70 (41.9)	22 (28.2)	48 (58.5)	<0.001
Lung metastasis		67 (39.9)	35 (44.3)	28 (34.1)	0.187
Brain metastasis		38 (22.6)	17 (21.5)	20 (24.4)	0.665
Bone metastasis		101 (60.1)	40 (50.6)	56 (68.3)	0.022
Lymph node metastasis		111 (66.1)	53 (67.1)	52 (63.4)	0.625
Treatment Line of T-DM1	1	7 (4.2)	5 (6.3)	2 (2.4)	0.035
	2	84 (50.0)	45 (57.0)	34 (41.5)	
	3	46 (27.4)	21 (26.6)	24 (29.3)	
	4	15 (8.9)	5 (6.3)	9 (11.0)	
	5	10 (6.0)	3 (3.8)	7 (8.5)	
	6	6 (3.6)	0 (0.0)	6 (7.3)	
Total number of treatment lines	Median (Min–Max)	4 (1–10)	3 (1–9)	4 (1–10)	0.006
Treatment of Pertuzumab before T-DM1	No	76 (45.2)	37 (46.8)	34 (41.5)	0.493
	Yes	92 (54.8)	42 (53.2)	48 (58.5)	
Treatment of Trastuzumab before T-DM1	No	11 (6.5)	6 (7.6)	5 (6.1)	0.707
	Yes	157 (93.5)	73 (92.4)	77 (93.9)	
Best Response to T-DM1	CR	18 (10.8)	15 (19.0)	3 (3.7)	0.004
	PR	68 (40.7)	35 (44.3)	30 (37.0)	
	SD	36 (21.6)	12 (15.2)	21 (25.9)	
	PD	45 (26.9)	17 (21.5)	27 (33.3)	
SUVmax	Median (Min–Max)	10 (1.9–78)	8.5 (1.9–78)	11.15 (2.5–31.1)	0.004
Albumin	Median (Min–Max)	40 (24.9–49)	40 (29–49)	40.35 (25.3–49)	0.725
CALA	Median (Min–Max)	118.3 (5.10–7054.60)	61.6 (5.1–116.4)	260.9 (118.3–7054.6)	<0.001
CRP	Median (Min–Max)	5.90 (0.2–121)	4.3 (0.2–121)	7.3 (0.2–111)	0.038
LDH	Median (Min–Max)	220.50 (106–1659)	196 (106–372)	254.5 (113–1659)	<0.001
CA 15-3	Median (Min–Max)	21.30 (2–1033)	11.8 (2.0–25.3)	46.5 (11.0–1033)	<0.001

Abbreviations: ECOG, Eastern Cooperative Oncology Group performance status; ER, estrogen receptor; PR, progesterone receptor; HER2, human epidermal growth factor receptor 2; BMI, body mass index; SUVmax, maximum standardized uptake value; T-DM1, trastuzumab emtansine; CR, complete response; PR, partial response; SD, stable disease; CRP, C-reactive protein; LDH, lactate dehydrogenase; CALA, (CA15-3 × LDH)/albumin; CA 15-3, cancer antigen 15-3.

**Table 2:** Comparison of adverse events between CALA groups.

	Total n (%)	CALA ≤ 118.3 n (%)	CALA > 118.3 n (%)	*p*-Value
Any adverse event				0.359
No	95 (56.9)	46 (58.2)	45 (54.9)	
Grade 1–2	65 (38.9)	28 (35.4)	35 (42.7)	
Grade 3–4	7 (4.2)	5 (6.3)	2 (2.4)	
Headache				0.888
Grade 1–2	15 (9.0)	7 (8.9)	7 (8.5)	
Grade 3–4	1 (0.6)	1 (1.3)	0 (0.0)	
Nausea/Vomiting				0.280
Grade 1–2	15 (9.1)	5 (6.5)	10 (12.3)	
Diarrhea				1.000
Grade 1–2	3 (1.8)	1 (1.3)	2 (2.4)	
Myalgia				0.079
Grade 1–2	34 (20.4)	21 (26.6)	12 (14.6)	
Hypertension				1.000
Grade 1–2	2 (1.2)	1 (1.3)	1 (1.2)	
Thrombocytopenia				0.437
Grade 1–2	21 (12.6)	9 (11.4)	11 (13.4)	
Grade 3–4	2 (1.2)	2 (2.5)	0 (0.0)	
Anemia				0.881
Grade 1–2	12 (7.2)	6 (7.6)	6 (7.3)	
Grade 3–4	1 (0.6)	1 (1.3)	0 (0.0)	
Neutropenia				0.648
Grade 1–2	16 (9.6)	10 (12.7)	6 (7.3)	
Grade 3–4	2 (1.2)	1 (1.3)	1 (1.2)	
Liver function test elevation				0.432
Grade 1–2	16 (9.6)	6 (7.6)	10 (12.2)	

Abbreviations: CALA, (CA15-3 × LDH)/albumin.

**Table 3:** Univariate and multivariate Cox regression analyses of overall survival.

Variables		Univariate		Multivariate	
		HR (95% CI Min–Max)	*p*-Value	HR (95% CI Min–Max)	*p*-Value
Age		0.997 (0.980–1.014)	0.703		
BMI		0.983 (0.949–1.018)	0.345		
Menopausal status		1.257 (0.855–1.847)	0.245		
ECOG			0.109		
	1 vs. 0	1.696 (1.114–2.582)	0.014		
	2 vs. 0	1.205 (0.602–2.413)	0.598		
	3 vs. 0	0.000	0.974		
Stage at Diagnosis			0.027		
	2	0.531 (0.258–1.092)	0.085		
	3	0.409 (0.192–0.871)	0.020		
	4	0.806 (0.424–1.535)	0.513		
Recurrence		0.603 (0.414–0.880)	0.009		
Tumor localization			0.742		
Left vs. right		1.026 (0.699–1.507)	0.896		
Bilateral vs. right		0.716 (0.283–1.806)	0.479		
ER status		0.770 (0.423–1.401)	0.392		
PR status		1.007 (0.620–1.636)	0.976		
Ki-67		0.997 (0.989–1.005)	0.444		
HER2 status		0.892 (0.581–1.371)	0.603		
Histologic subtype			0.411		
invasive lobular		0.796 (0.196–3.229)	0.750		
other		0.466 (0.147–1.473)	0.193		
Liver metastasis		2.047 (1.397–2.999)	<0.001	1.495 (0.970–2.305)	0.068
Lung metastasis		1.344 (0.927–1.949)	0.119	1.509 (1.012–2.250)	0.044
Brain metastasis		1.752 (1.164–2.637)	0.007	1.536 (0.964–2.449)	0.071
Bone metastasis		1.527 (1.032–2.259)	0.034	1.309 (0.845–2.028)	0.228
Lymph node metastasis		0.734 (0.498–1.083)	0.119		
Treatment of Pertuzumab before T-DM1		0.960 (0.658–1.400)	0.832		
Treatment of Trastuzumab before T-DM1		1.384 (0.673–2.846)	0.377		
Best Response to T-DM1			<0.001		
	PR	2.008 (0.893–4.515)	0.092		
	SD	3.330 (1.444–7.679)	0.005		
	PD	8.093 (3.569–18.349)	<0.001		
Treatment line of T-DM1		1.121 (0.960–1.309)	0.148		
Total number of treatment line		0.943 (0.859–1.035)	0.215		
SUVmax		0.999 (0.975–1.024)	0.954		
Albumin		0.936 (0.896–0.979)	0.003		
CRP		1.009 (1.001–1.017)	0.034	1.008 (1.000–1.016)	0.045
Lymphocyte		1.000 (1.000–1.000)	0.028	1.000 (1.000–1.000)	0.292
LDH		1.002 (1.001–1.003)	0.001		
CALA		1.271(1.099–1.471)	0.001		
CALA > 181.3		1.699(1.151–2.506)	0.008	1.671 (1.088–2.565)	0.019
CA 15-3		1.001 (1.000–1.002)	0.034		
Adverse effect			0.008		0.010
Grade 1–2		0.537 (0.359–0.803)	0.002	0.515 (0.331–0.803)	0.003
Grade 3–4		1.040 (0.451–2.399)	0.927	1.224 (0.512–2.923)	0.649

Abbreviations: HR, hazard ratio; CI, confidence interval; ECOG, Eastern Cooperative Oncology Group performance status; ER, estrogen receptor; PR, progesterone receptor; HER2, human epidermal growth factor receptor-2; BMI, body mass index; SUVmax, maximum standardized uptake value; T-DM1, trastuzumab emtansine; PR, partial response; SD, stable disease; CRP, C-reactive protein; LDH, lactate dehydrogenase; CALA, (CA15-3 × LDH)/albumin; CA 15-3, cancer antigen 15-3.

**Table 4:** Univariate and multivariate Cox regression analyses of progression-free survival.

Variables	Univariate		Multivariate	
	HR (95% CI Min–Max)	*p*-Value	HR (95% CI Min–Max)	*p*-Value
**Age**	0.994 (0.980–1.009)	0.436		
**BMI**	0.987 (0.963–1.013)	0.326		
**Menopausal Status**	1.059 (0.755–1.487)	0.738		
**ECOG**		0.634		
**1 vs. 0**	1.257 (0.863–1.830)	0.234		
**2 vs. 0**	1.268 (0.676–2.377)	0.459		
**3 vs. 0**	0.000	0.968		
**Stage at Diagnosis**		0.612		
**2**	0.862 (0.445–1.670)	0.659		
**3**	0.707 (0.354–1.409)	0.324		
**4**	0.948 (0.512–1.755)	0.864		
**Recurrence**	0.832 (0.597–1.159)	0.278		
**Tumor localization**		0.568		
**Left vs. right**	1.061 (0.751–1.499)	0.737		
**Bilateral vs. right**	1.538 (0.695–3.402)	0.288		
**ER status**	0.872 (0.526–1.446)	0.595		
**PR status**	1.041 (0.683–1.587)	0.851		
**Ki-67**	0.996 (0.989–1.003)	0.299		
**HER2 status**	0.762 (0.524–1.107)	0.154		
**Histologic subtype**		0.632		
**invasive lobular**	0.622 (0.154–2.519)	0.506		
**other**	0.699 (0.258–1.895)	0.482		
**Liver metastasis**	1.238 (0.883–1.735)	0.215	1.246 (0.852–1.824)	0.257
**Lung metastasis**	1.222 (0.871–1.715)	0.246	1.293 (0.905–1.847)	0.159
**Brain metastasis**	1.134 (0.770–1.671)	0.524	1.260 (0.834–1.903)	0.273
**Bone Metastasis**	1.250 (0.887–1.763)	0.202		
**Lymph node metastasis**	1.048 (0.735–1.495)	0.796		
**Treatment of Pertuzumab before T-DM1**	1.206 (0.863–1.684)	0.272		
**Treatment of Trastuzumab before TD-M1**	2.010 (1.017–3.973)	0.045	1.230 (0.604–2.503)	0.568
**Best Response to TD-M1**		<0.001		
**PR**	2.352 (1.152–4.800)	0.019		
**SD**	5.460 (2.569–11.604)	<0.001		
**PD**	20.770 (9.830–43.889)	<0.001		
**Treatment line of T-DM1**	1.073 (0.941–1.222)	0.292		
**Total number of treatment line**	1.164 (1.072–1.265)	<0.001	1.151 (1.047–1.267)	0.004
**SUVmax**	1.015 (0.995–1.035)	0.150		
**A** **l** **bumin**	0.991 (0.952–1.033)	0.682		
**CRP**	1.008 (1.000–1.015)	0.040	1.007 (0.999–1.014)	0.080
**Lymphocyte**	1.000 (1.000–1.000)	0.190		
**LDH**	1.001 (1.000–1.002)	0.048		
**CALA**	1.236 (1.084–1.409)	0.002		
**CALA > 181.3**	1.401 (1.004-1.956)	0.047	1.112 (0.767–1.613)	0.576
**CA15** **-** **3**	1.001 (1.000–1.002)	0.048		
**Adverse effect**		0.311		
**Grade 1–2**	0.854 (0.607–1.200)	0.364		
**Grade 3–4**	0.499 (0.182–1.366)	0.176		

Abbreviations: HR, hazard ratio; CI, confidence interval; ECOG, Eastern Cooperative Oncology Group performance status; ER, estrogen receptor; PR, progesterone receptor; HER2, human epidermal growth factor receptor 2; BMI, body mass index; SUVmax, maximum standardized uptake value; T-DM1, trastuzumab emtansine; PR, partial response; SD, stable disease; CRP, C-reactive protein; LDH, lactate dehydrogenase; CALA, (CA15-3 × LDH)/albumin; CA 15-3, cancer antigen 15-3.

## Data Availability

The original contributions presented in this study are included in the article. Further inquiries can be directed to the corresponding authors.

## Abbreviations

The following abbreviations are used in this manuscript:

HER-2	Human Epidermal Growth Factor Receptor-2
T-DM1	Trastuzumab Emtansine
LDH	Lactate Dehydrogenase
CA15-3	Cancer Antigen 15-3
CALA	CA15-3 × LDH/Albumin
OS	Overall Survival
PFS	Progression-Free Survival
ROC	Receiver Operating Characteristic
AUC	Area Under the Curve
CI	Confidence Interval
HR	Hazard Ratio
AIC	Akaike Information Criterion
NLR	Neutrophil-to-Lymphocyte Ratio
PLR	Platelet-to-Lymphocyte Ratio
CRP	C-Reactive Protein
HALP	Hemoglobin, Albumin, Lymphocyte, Platelet Score
CALLY	CRP–Albumin–Lymphocyte Index
dNLR	Derived Neutrophil-to-Lymphocyte Ratio
PNI	Prognostic Nutritional Index
AAPR	Albumin-to-Alkaline Phosphatase Ratio
